# Psychological barriers in future food gatekeepers: How germ aversion and perceived infectability mediate nutrition-related food neophobia among pre-service preschool teachers

**DOI:** 10.3389/fnut.2026.1863520

**Published:** 2026-07-10

**Authors:** Lu Xing, Xueqi Wu

**Affiliations:** 1 School of Preschool Education, Changsha Normal University, Changsha, China; 2 School of Education, Faculty of Social Sciences and Leisure Management, Taylor's University, Subang Jaya, Malaysia; 3 Taylor's Culinary Institute, Faculty of Social Sciences and Leisure Management, Taylor's University, Subang Jaya, Malaysia

**Keywords:** food disgust, germ aversion, nutrition-related food Neophobia, perceived infectability, pre-service teachers

## Abstract

**Background:**

The development of nutritional health literacy in early childhood is structurally influenced by adult food gatekeepers. Approached as a sociological study of food, this research investigates the relationship between Nutrition-Related Food Neophobia (NRFN) and Food Disgust (FD) among pre-service preschool teachers, examining the mediating roles of the Behavioral Immune System—specifically Perceived Infectability (PI) and Germ Aversion (GA)—as underlying behavioral determinants.

**Methods:**

A cross-sectional survey was conducted among 548 pre-service preschool teachers (males 43.2%, females 56.8%) in Changsha, China. Participants completed validated scales including the Perceived Vulnerability to Disease Questionnaire, the Nutrition-Related Food Neophobia Scale, and the Food Disgust Scale, evaluating biofortified rice as a nutritional stimulus. Data were analyzed using a two-step Structural Equation Modeling (SEM) approach.

**Results:**

SEM analysis revealed that NRFN was significantly and positively associated with FD. PI and GA collectively mediated this relationship, explaining 54.69% of the total effect. The mediating influence of GA (30.98%) was numerically larger than that of PI (23.71%), indicating that the hygiene training of future educators may inadvertently contribute to their affective, evolutionary resistance to novel nutritional foods over their cognitive immune assessments.

**Conclusion:**

Despite the formal health and nutritional training anticipated in pre-service educators, our findings suggest that evolutionary pathogen-avoidance mechanisms continue to be strongly associated with their food aversion. These psychological barriers can potentially limit the implementation fidelity of nutritional educational tools within the kindergarten’s social space of food. Enhancing children’s dietary diversity and health literacy could benefit from shifting preventive strategies upstream, integrating food sociology and evolutionary psychology into teacher-training programs to dismantle germ aversion and build psychological resilience.

## 1 Introduction

The establishment of nutritional health literacy during early childhood is a critical preventive strategy against malnutrition and obesity. While extensive research has mapped children’s dietary behaviors, the structural and environmental determinants shaping these habits are fundamentally overseen by adult “food gatekeepers.” First conceptualized by Lewin in 1943 to describe those who control the channels through which food reaches the table, the gatekeeper theory underscores that preschool teachers act as the primary nutritional gatekeepers in early childhood education settings. The kindergarten dining environment operates as a “social space of food” (1), where children internalize dietary norms, cultural acceptability, and nutritional habits. Therefore, the effectiveness of any nutritional educational tools deployed in kindergartens relies heavily on the psychological receptiveness of these educators.

Currently, societies undergoing rapid socio-economic transitions—such as China—are experiencing a state of “compressed modernity” (2, 3). In the realm of food systems, this entails the rapid collision of deeply entrenched traditional agrarian dietary norms with highly advanced, industrialized nutritional sciences (e.g., biofortification) within a highly condensed timeframe. This rapid transition leaves little time for cultural adaptation, creating a structural tension. In cities like Changsha, regional culinary traditions are exceptionally strong, and traditional staples like rice are not merely sources of calories but are revered as natural, culturally sacred symbols that anchor communal identity and emotional security.

When modern nutritional sciences introduce technologically altered foods, such as biofortified rice, into this context, it triggers a cognitive conflict. Within the “social space of food,” these nutritional interventions are often subconsciously perceived not as health upgrades, but as a “medicalized invasion” that alienates the food from its natural and cultural roots. This tension significantly exacerbates psychological barriers. Future food gatekeepers (pre-service teachers) face this conflict directly. They have received formal training in modern hygiene and nutritional science, yet their underlying cultural schemas remain deeply traditional. This structural dissonance disrupts their internal sense of food safety, causing their personal food acceptance mechanisms to act as powerful, hidden behavioral determinants. As a result, this cultural and technological friction may increase their apprehension toward nutritionally modified foods, potentially limiting rather than expanding the children’s future dietary environment.

Food neophobia, defined as the reluctance to eat or avoid new foods, is a well-documented evolutionary trait. In examining food rejection, Rozin and Fallon (4) established that disgust is primarily a food-related emotion driven by the fear of incorporating an offensive or contaminated substance. Building on this, Tybur et al. (5) demonstrated that pathogen disgust and Germ Aversion (GA) function as evolved psychological disease-avoidance mechanisms. Recent evidence further indicates that these disease-avoidance motivations are intimately connected to the manifestation of food neophobia (6). Despite receiving formal education in preschool hygiene and nutrition, pre-service teachers may still experience Food Disgust (FD) when confronted with nutritionally altered foods, as their evolutionary pathogen avoidance systems override their cognitive nutritional knowledge.

In this context, Perceived Infectability (PI)—an individual’s chronic belief regarding their vulnerability to illness—and Germ Aversion—the emotional discomfort caused by potential environmental pathogens—emerge as critical behavioral determinants. If pre-service teachers reject nutritionally fortified foods due to underlying GA and PI, their capacity to implement responsive feeding practices and utilize novel nutritional educational tools for children could be compromised. While current preventive strategies for pediatric food neophobia predominantly employ ‘downstream’ interventions—such as repeated exposure, sensory education (e.g., the Sapere method), and flavor-flavor learning directly targeting children—these methods often exhibit inconsistent long-term efficacy. A critical, yet overlooked, factor is the ‘implementation fidelity’ of these strategies, which is inherently mediated by the psychological state of the educators. By shifting the analytical focus from the child to the ‘upstream’ gatekeeper, this study seeks to uncover the behavioral determinants that govern the micro-food environment of the kindergarten. This study examines the relationships among Nutrition-Related Food Neophobia, Food Disgust, Perceived Infectability, and Germ Aversion among pre-service preschool teachers. We propose the following hypotheses:

### 1.1 Nutrition-related food neophobia and food disgust

The “omnivore’s dilemma” posits that humans must constantly balance the drive to seek novel nutrients against the risk of ingesting toxins (4). To navigate this dilemma, evolutionary perspectives propose that Food Neophobia (FN) may function as a primary cognitive defense mechanism. FN represents a generalized reluctance to consume unfamiliar foods (7). In modern food systems, this neophobia has evolved beyond mere sensory unfamiliarity into Nutrition-Related Food Neophobia (NRFN)—a specific resistance triggered by unfamiliar nutritional profiles or biofortification processes (8).

While NRFN serves as the cognitive appraisal of novelty, Food Disgust (FD) operates as the profound affective and physiological rejection response. Rozin and Fallon (4) established that disgust is inherently a food-related emotion designed to prevent the incorporation of potentially offensive substances into the body. When future food gatekeepers (i.e., pre-service teachers) encounter nutritionally altered foods, the cognitive unfamiliarity (NRFN) disrupts their established “social space of food” and safe-food schemas (1). This cognitive disruption translates directly into an affective defense mechanism. Previous empirical studies indicate that fear of negative somatic consequences is a primary driver of core disgust (9). Therefore, a strong cognitive aversion to novel nutritional attributes is expected to be strongly associated with a visceral disgust response.

*H1*: Nutrition-related Food Neophobia is positively associated with Food Disgust.

### 1.2 The mediating role of perceived infectability

Understanding the pathway from NRFN to FD requires examining the underlying psychological mechanisms of disease avoidance. The Behavioral Immune System (BIS) theory suggests that humans possess a suite of psychological mechanisms to detect and avoid cues associated with infectious pathogens (10). Within this system, Perceived Vulnerability to Disease (PVD) captures individual differences in these defensive responses, comprising two distinct dimensions: Perceived Infectability (PI) and Germ Aversion (11).

Perceived infectability refers to an individual’s conscious, cognitive belief regarding their own immunological susceptibility to infectious diseases. When individuals with high NRFN are presented with novel, nutritionally fortified foods, they lack the historical and cultural safety verification traditionally associated with familiar diets. In a society experiencing “compressed modernity,” where food technologies advance faster than cultural adaptation (2, 3), this lack of familiarity acts as a psychological stressor. The uncertainty surrounding the physiological impact of the novel food heightens the individual’s subjective sense of immunological vulnerability (PI). Consequently, once this belief of high personal vulnerability is activated, the individual is highly likely to evaluate the novel food as a physiological threat, thereby triggering severe Food Disgust (FD) as a final behavioral barrier to ingestion.

*H2a*: Nutrition-related Food Neophobia is positively associated with Perceived Infectability.

*H2b*: Perceived Infectability is positively associated with Food Disgust.

*H2*: Perceived Infectability positively mediates the relationship between Nutrition-related Food Neophobia and Food Disgust.

### 1.3 The mediating role of germ aversion

While PI is a cognitive belief about personal immunity, Germ Aversion operates as an affective, visceral discomfort elicited by situations or stimuli perceived to facilitate pathogen transmission (5, 11). This distinction is particularly crucial for pre-service preschool teachers. Through formal education in early childhood hygiene and health, these future gatekeepers have internalized rigorous sanitation norms.

**Figure 1:**
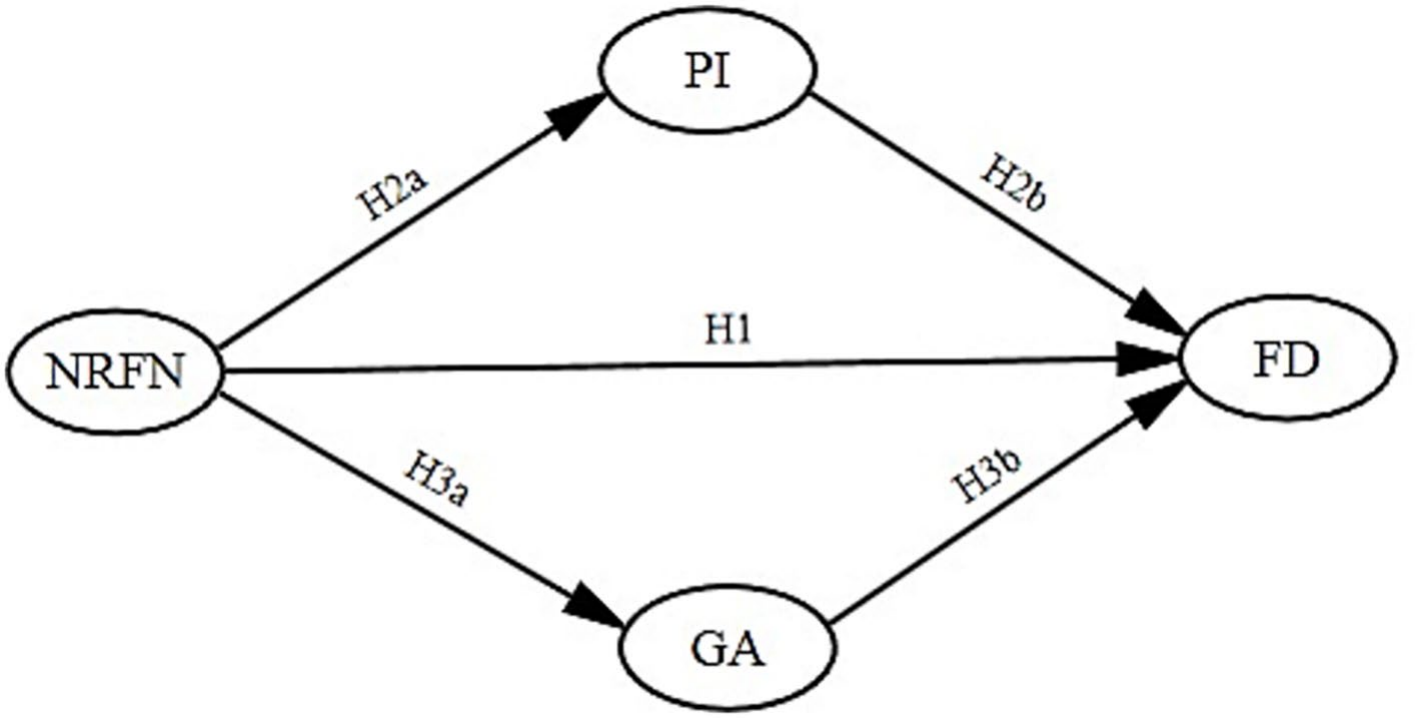
Conceptual model of the hypothesized relationships. NRFN, Nutrition-related Food Neophobia; PI, Perceived Infectability; GA, Germ Aversion; FD, Food Disgust.

When confronted with nutritionally novel foods (high NRFN), the departure from traditional, visually recognizable food forms often registers as “unnatural.” Evolutionary psychology dictates that deviations from natural or familiar food states are often heuristically processed as signs of spoilage or contamination (12). For health-trained gatekeepers, the cognitive uncertainty of NRFN specifically activates their deeply ingrained Germ Aversion. The novel food is implicitly categorized as a potential vector for contamination, conflicting with their professional and personal hygiene schemas. This intense affective discomfort (GA) then acts as a powerful catalyst, potentially translating the initial neophobia into profound Food Disgust (FD) to ensure avoidance. Therefore, GA serves as a critical affective bridge between the fear of nutritional novelty and ultimate food rejection (Figure 1).

*H3a*: Nutrition-related Food Neophobia is positively associated with Germ Aversion.

*H3b*: Germ Aversion is positively associated with Food Disgust.

*H3*: Germ Aversion positively mediates the relationship between Nutrition-related Food Neophobia and Food Disgust.

## 2 Materials and methods

### 2.1 Measures

All instruments were adapted from established scales with proven reliability and validity. Through discussions with experts and scholars, we modified and optimized some scales to eliminate problems that were not consistent with the topic of this research, with these measures used to adapt to the current research background (Table 1).

**Table 1:** Measurement model, descriptive statistics.

Constructs	Variable	Unstd.	S. E.	T-Value	P	Std.	SMC	CR	AVE	Cronbach’s alpha
Nutrition-related food neophobia (NRFN) (8)								0.886	0.612	0.883
	I am afraid that this rice will harm my health.	1.000				0.889	0.790			
	I will try this rice only after I have made sure it is safe for consumption.	0.649	0.039	16.541	***	0.636	0.404			
	I am not willing to try this rice because I think ordinary rice has all the micronutrients I need.	0.863	0.036	24.047	***	0.818	0.669			
	I am willing to try this rice even if that makes me feel uncomfortable. [R]	0.809	0.043	18.865	***	0.699	0.489			
	I am not willing to try this rice because I fear that more than the naturally occurring amount of micronutrient in rice is not suitable for my body.	0.928	0.037	25.107	***	0.840	0.706			
Perceived Infectability (PI) (13)								0.887	0.530	0.886
	If an illness is ‘going around’, I will get it.	1.000				0.754	0.569			
	My past experiences make me believe I am not likely to get sick even when my friends are sick. [R]	0.981	0.059	16.499	***	0.716	0.513			
	I have a history of susceptibility to infectious disease.	0.997	0.059	16.964	***	0.734	0.539			
	In general, I am very susceptible to colds, flu and other infectious diseases.	0.913	0.060	15.162	***	0.662	0.438			
	I am more likely than the people around me to catch an infectious disease.	0.939	0.059	15.866	***	0.690	0.476			
	I am unlikely to catch a cold, flu or other illness, even if it is ‘going around’. [R]	0.973	0.055	17.839	***	0.770	0.593			
	My immune system protects me from most illnesses that other people get. [R]	0.965	0.055	17.623	***	0.761	0.579			
Germ aversion (GA) (13)								0.859	0.468	0.857
	It really bothers me when people sneeze without covering their mouths.	1.000				0.643	0.413			
	I am comfortable sharing a water bottle with a friend. [R]	1.089	0.081	13.521	***	0.693	0.480			
	I do not like to write with a pencil someone else has obviously chewed on.	0.944	0.074	12.809	***	0.648	0.420			
	I prefer to wash my hands pretty soon after shaking someone’s hand.	1.020	0.080	12.729	***	0.643	0.413			
	I dislike wearing used clothes because you do not know what the last person who wore it was like.	1.282	0.084	15.291	***	0.822	0.676			
	My hands do not feel dirty after touching money. [R]	1.108	0.082	13.578	***	0.697	0.486			
	It does not make me anxious to be around sick people. [R]	1.055	0.085	12.430	***	0.624	0.389			
Food disgust (FD) (15)								0.905	0.616	0.904
	To see raw meat.	1.000				0.787	0.619			
	To eat with dirty silverware in a restaurant.	1.021	0.052	19.817	***	0.787	0.619			
	A meal prepared by a cook who has greasy hair and dirty fingernails.	1.027	0.059	17.296	***	0.704	0.496			
	If the cook in a restaurant has an open cut.	0.908	0.052	17.413	***	0.708	0.501			
	To eat raw fish like sushi.	1.141	0.053	21.397	***	0.836	0.699			
	Food donated from a neighbor whom I barely know.	1.185	0.053	22.568	***	0.874	0.764			

SE, standard Error; P, *p*-value; SMC, squared multiple correlations; CR, composition reliability; AVE, average variance extracted. ***p < 0.001; *N* = 548. NRFN = Nutrition-related Food Neophobia; PI = Perceived Infectability; GA = Germ Aversion; FD = Food Disgust. [R] = indicates reverse score items.

The Perceived Vulnerability to Disease Questionnaire (PVD) is a validated scale (11). Subsequently, Li et al. (13) conducted a cross-cultural translation and further verified its validity in a Chinese context. We utilized this 14-item Chinese version, which removed one temporally and culturally inconsistent item (Item 15: “I avoid using public telephones because of the risk that I may catch something from the previous user”) from the original 15-item scale. Considering the contemporary context where public telephones are virtually obsolete, this specific item lacks ecological validity and relevance for modern university students. Its removal, consistent with adaptations for current Chinese populations (13), ensures optimal reliability and validity for our sample. Respondents used a 7-point Likert scale (completely disagree to completely agree). The scale is composed of two dimensions, perceived infectability and germ aversion. The Cronbach’s alpha for the PI part is 0.886, and for the GA part is 0.857.

**Figure 2:**
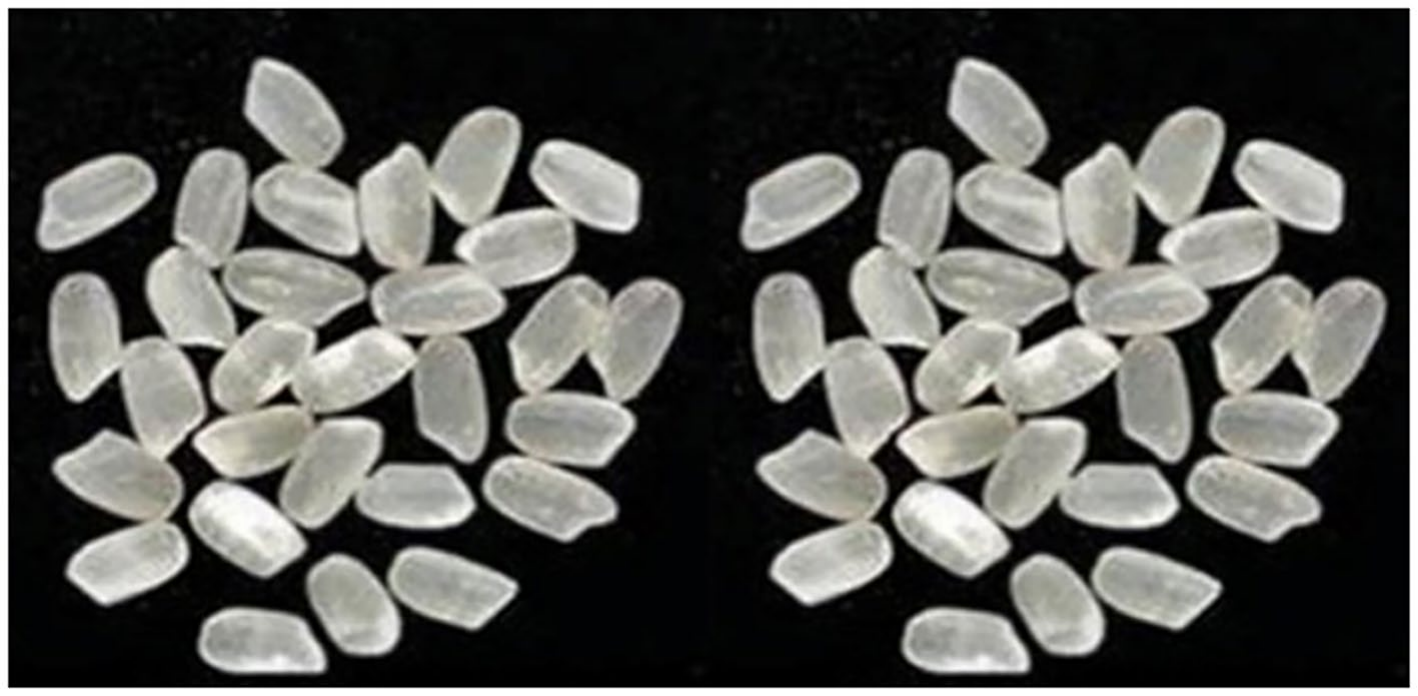
Visual stimulus used in the Nutrition-Related Food Neophobia Scale. Regular rice is shown on the left, and nutritionally fortified rice is shown on the right. Adapted from Razzaq et al. (8), licensed under CC BY 4.0.

The Nutrition-Related Food Neophobia Scale (NRFNS) originally evolved from the Food Neophobia Scale developed by Pliner and Hobden (7), Amar Razzaq et al. (8) separated and examined nutrition-related dimensions. It consists of 5 items on a 5-point Likert scale used by participants as a measure of Nutrition-Related Food Neophobia. To ensure the linguistic equivalence and cultural validity of the NRFNS within the Chinese socio-cultural context, a standard forward-backward translation procedure was employed (14). Two bilingual researchers independently translated the original English items into Chinese, and a third expert back translated them into English to resolve any conceptual discrepancies, ensuring the tool accurately captures the behavioral determinants of food choice in a Chinese setting. To elicit a realistic neophobic response, a specific visual stimulus depicting a hypothetical nutritional enhancement scenario was utilized. Rice was selected as the target food because it is the fundamental staple in the Southern Chinese diet, representing a deeply entrenched “social space of food.” Introducing a biofortified, technologically altered version of this highly familiar staple serves as an effective instrument to disrupt cultural schemas and accurately assess the underlying behavioral determinants of food rejection. Before answering these five items, a picture (Figure 2) was shown and participants were informed: “Suppose these are the two kinds of rice you see in the supermarket. The white rice on the left is ordinary rice, and the right rice contains more nutrients; biologists have used some techniques to double the trace elements contained in rice. So far, there are no reports of other people having adverse reactions from eating the right rice. Please express your opinion on the right rice, answer the questions on the five items.”

It is important to clarify that this instruction was designed as a hypothetical nutritional enhancement scenario aimed at assessing generalized cognitive reactions to food technology (i.e., biofortification). The phrasing “used some techniques” was employed to elicit a baseline neophobic response to unnaturalness, but it does not represent a specific Genetically Modified Organism (GMO). We acknowledge that the ambiguity of this phrasing might conflate general nutritional enhancement with specific genetic modification concerns, which was addressed as a potential constraint on construct validity in the limitation section.

The Cronbach’s alpha for the Nutrition-Related Food Neophobia Scale calculated for the study sample was 0.883.

Food Disgust Scale (FDS) original designers of this subscale were Hartmann and Siegrist. After cross-cultural revision by Zhang Lu’s team at Inner Mongolia Normal University, a version adapted to Chinese university students was obtained (15). This version of the scale is composed of 6 items, which are used to measure food disgust, and the 5-point Likert scale is used as the measure of food disgust. In this study, the FDS was employed to capture a general, trait-like propensity for food disgust (e.g., toward raw meat or dirty silverware), rather than disgust toward the specific biofortified rice stimulus. This distinction is theoretically crucial: while the biofortified rice was utilized as a situational nutritional trigger to elicit cognitive unfamiliarity (NRFN), the FDS assesses how this specific cognitive disruption is associated with the activation of the individual’s broader, underlying physiological disgust sensitivity. Cronbach’s alpha calculated for the study sample was 0.904.

Minor modifications were made based on pilot feedback from three experts in teacher education to enhance content validity. PI and GA were rated on a 7-point Likert scale ranging from 1 (completely disagree) to 7 (completely agree). FD and NRFN were rated on a 5-point Likert scale ranging from 1 (completely disagree) to 5 (completely agree). This study’s data was collected in Chinese. For accuracy, a Chinese - English parallel version is provided in the Supplementary material.

### 2.2 Research setting and participants

This study employed a cross-sectional survey design. Participants were recruited using purposive sampling from pre-service preschool education students at Changsha Normal University in Hunan Province, China, as this cohort represents the future primary food gatekeepers within kindergarten settings. The data collection period spanned from February 2024 to March 2024. Participants agreed to participate in the study before starting and could interrupt at any time. Participants were informed that they would not receive payment/reward for taking this survey. The researchers presented them with the following instructions, “The researcher will conduct a study on preschool education pre-service teachers’ Food Neophobia, Perceived Infectability, Food Disgust and Germ Aversion. The respondents will have the opportunity to express their opinions on the consumer attitudes which addressed in the question. Because there is no right or wrong answer, and the questionnaire is anonymous, as well as the data collection will be used for statistical purposes in a comprehensive form.” Subsequently, the respondents were asked to take the scale test. Finally, researcher collected participant demographic information.

In China, pre-service teachers often refer to college students majoring in teacher training. The respondents were 589 college students at normal universities, aged over 18 and under 22. All participants were over 18 years of age, and no minors were involved. Twenty-one people (3.57%) did not complete the questionnaire and were therefore excluded. Then 20 questionnaires with the same answers to all questions were removed. Finally, 548 valid questionnaires were obtained, of which 237 were answered by males (43.2%) and 311 were answered by females (56.8%). This final sample size of 548 extensively exceeds the recommended minimum requirements for structural equation modeling. According to the “rule of 10” (requiring at least 10 participants per estimated parameter) or the absolute minimum threshold of 200 cases for complex models (16), the sample size provides robust statistical power to validate these psychological barriers and behavioral determinants.

The study was conducted in accordance with the Declaration of Helsinki. The protocol was reviewed and approved by the Ethics Committee of Changsha Normal University (Protocol code:2023–31).

### 2.3 Data analysis

Data analysis was performed using SPSS 26.0 and AMOS 26.0. We adopted a two-step structural equation modeling (SEM) approach (17) to test the hypotheses.

#### 2.3.1 Common method bias assessment

Given the use of self-reported cross-sectional data, Common Method Bias (CMB) was assessed. Harman’s single-factor test was conducted using SPSS to evaluate whether a single general factor accounted for the majority of the covariance among the measures.

#### 2.3.2 Measurement model assessment

Confirmatory factor analysis (CFA) was first conducted to evaluate the reliability and validity of the measurement model. Internal consistency was assessed using Cronbach’s alpha (>0.70) and Composite Reliability (CR > 0.70). Convergent validity was evaluated using the Average Variance Extracted (AVE > 0.50) and standardized factor loadings (>0.50) (18, 19). Discriminant validity was examined using the Fornell-Larcker criterion, which requires the square root of the AVE for each construct to exceed its correlations with other constructs (20).

#### 2.3.3 Structural model assessment

The fit of the structural model was evaluated using the Maximum Likelihood (ML) estimation method. Model fit was assessed based on standard indices: Chi-square/df ratio (<3.0), CFI (>0.90), TLI/NFI (>0.90), RMSEA (<0.08), and SRMR (<0.08) (21, 22).

#### 2.3.4 Mediation analysis

To test the mediating effects (indirect paths), we utilized the bootstrapping method with 5,000 resamples. The 95% bias-corrected confidence intervals (CI) were calculated. An indirect effect is considered statistically significant if the 95% CI does not contain zero (23, 24). Unstandardized path coefficients were utilized to calculate the indirect effects and their corresponding mediation ratios.

## 3 Results

### 3.1 Measurement model assessment

To strictly monitor Common Method Bias (CMB), we employed the Harman’s single-factor test. The results indicated that the first principal component 39.36%, explained less than the recommended 50% threshold of the total variance. This suggests that common method bias may not be a pervasive issue in this dataset. This step provides initial support for the validity of the subsequent CFA and structural models.

Before testing the structural relationships, a Confirmatory Factor Analysis (CFA) was conducted using the Maximum Likelihood method (25) to assess the reliability and validity of the constructs. The four constructs of the model were Nutrition-related Food Neophobia, Germ aversion, Perceived infectability, and Food disgust.

As shown in Table (Table 1), the factor loadings for all items ranged from 0.624 to 0.889, exceeding the recommended threshold of 0.501. The Cronbach’s alpha and Composite Reliability (CR) values for all constructs were greater than 0.80, well above the 0.70 benchmark, indicating high internal consistency. Furthermore, the Average Variance Extracted (AVE) values range from 0.468 for GA, while others are between 0.530 and 0.612, exceeding the 0.50. Fornell and Larcker’s (20) research indicates that if the composite reliability (CR) value meets the standard (usually >0.7), even if AVE was slightly below 0.5, the convergent validity was still acceptable, as CR was a more stable reliability indicator, which supports adequate convergent validity.

Discriminant validity was assessed using the Fornell-Larcker criterion (20). As presented in Table 2, the square root of the AVE for each construct (shown in bold on the diagonal) was greater than its highest correlation with any other construct. This confirms that the constructs are empirically distinct. Additionally, Table 2 presents the descriptive statistics and bivariate correlation coefficients among all latent variables, providing an intuitive overview of their interrelationships.

**Table 2:** Analysis of discriminant validity (20).

Constructs	Mean	SD	GA	PI	FD	NRFN
GA	4.233	1.601	**0.684**			
PI	4.144	1.610	0.594	**0.728**		
FD	4.087	1.139	0.531	0.507	**0.785**	
NRFN	3.644	1.182	0.608	0.652	0.535	**0.782**

1. Square root of AVE in bold on diagonals. 2. Off diagonals are Pearson correlation of constructs. NRFN = Nutrition-related Food Neophobia; PI = Perceived Infectability; GA = Germ Aversion; FD = Food Disgust.

### 3.2 Structural model test

The structural model was evaluated using Maximum Likelihood (ML) estimation. The fit indices indicated that the hypothesized model fits the data well (Table 3): χ2 = 598.396, df = 270, χ2/df = 2.216(<3.0) CFI = 0.956 (>0.90), NFI = 0.922 (>0.90), RMSEA = 0.047 (<0.08), and SRMR = 0.058(<0.08). These indices suggest the theoretical model aligns well with the observed data.

**Table 3:** Model fitting index.

Fit indices	χ2	df	χ2/df	GFI	RMSEA	CFI	NFI	AGFI	SRMR
Reference value	–	–	<3	>0.9	<0.08	>0.9	>0.9	>0.9	<0.08
Model value	598.396	270.000	2.216	0.918	0.047	0.956	0.922	0.901	0.058

χ^2^, Chi-Square Minimum Fit Function; DF, Degrees of Freedom; χ2/DF, Chi-Square to Degrees of Freedom ratio; GFI, Goodness of Fit Index; AGFI, Adjusted Goodness of Fit Index; CFI, Comparative Fit Index; RMSEA, Root Mean Square Error of Approximation; NFI, Normed Fit Index; SRMR, Standardized Root Mean Square Residual.

### 3.3 Hypothesis testing (direct effects)

The path coefficients and significance levels for the direct effects are summarized in Table 4. To facilitate the comparison of relative effect sizes across different paths, standardized path coefficients (*β*) are reported in this section.

**Table 4:** Hypothesis testing.

Hypotheses	Construct	Unstandardized path coefficient	S.E.	C.R.	*P*	Standardized path coefficient	Results
H2a	NRFN→PI	0.754	0.052	14.378	***	0.669	Supported
H3a	NRFN→GA	0.584	0.050	11.750	***	0.627	Supported
H2b	PI→FD	0.135	0.040	3.356	***	0.194	Supported
H3b	GA → FD	0.225	0.048	4.707	***	0.269	Supported
H1	NRFN→FD	0.193	0.054	3.566	***	0.247	Supported

***p < 0.001. NRFN = Nutrition-related Food Neophobia; PI = Perceived Infectability; GA = Germ Aversion; FD = Food Disgust.

First, Nutrition-Related Food Neophobia was significantly and positively associated with Perceived Infectability (*β* = 0.669, *p* < 0.001) and Germ Aversion (*β* = 0.627, *p* < 0.001), supporting H2a and H3a.

Second, Perceived infectability was positively associated with Food Disgust (FD) (*β* = 0.194, *p* < 0.001), supporting H2b.

Third, Germ Aversion (GA) was positively associated with Food Disgust (FD) (*β* = 0.269, *p* < 0.001), supporting H3b.

Finally, Nutrition-Related Food Neophobia maintained a significant direct effect on Food Disgust (*β* = 0.247, *p* < 0.001) even after accounting for the mediators, supporting H1.

Collectively, these results suggest that Nutrition-Related Food Neophobia is not only directly associated with Food Disgust, but these findings also provide preliminary support for the possibility that “Perceived Infectability” and “Germ Aversion” may play a mediation role.

### 3.4 Mediation analysis (indirect effects)

To test the mediating roles of Perceived Infectability and Germ Aversion (GA), we employed the bootstrapping method with 5,000 resamples and 95% bias-corrected confidence intervals (CI). In accordance with standard mediation analysis practices, unstandardized coefficients (*B*) are reported for all indirect, direct, and total effects in this section to accurately calculate the mediation ratios. The results of the mediation analysis are presented in Table 5.

**Table 5:** Mediation analysis.

SIE	Point estimate	Product of coefficients		Bootstrapping = 5,000				Ratio %
				Bias-Corrected 95%CI		Percentile 95%CI		
		SE	*Z*	Lower	Upper	Lower	Upper	
Indirect effect								
NRFN-PI-FD	0.101	0.040	2.525	0.030	0.188	0.026	0.181	23.71%
NRFN-GA-FD	0.132	0.037	3.568	0.067	0.215	0.064	0.210	30.98%
Total	0.233	0.050	4.660	0.141	0.335	0.137	0.332	54.69%
Direct effect								
NRFN-FD	0.193	0.061	3.164	0.077	0.317	0.077	0.317	45.31%
Total effect								
NRFN-FD	0.426	0.049	8.694	0.328	0.520	0.326	0.519	100%
Model without mediators								
NRFN-FD	0.420	0.050	8.400					

1. Unstandardized estimating of 5,000 bootstrap sample. All reported estimates for indirect, direct, and total effects are unstandardized coefficients. The Ratio % (percentage mediated) was calculated by dividing the unstandardized indirect effect by the unstandardized total effect. 2. ***p < 0.001. NRFN = Nutrition-related Food Neophobia; PI = Perceived Infectability; GA = Germ Aversion; FD = Food Disgust.

#### 3.4.1 Indirect effect via perceived infectability (H2)

The indirect path from Nutrition-Related Food Neophobia to Food Disgust via Perceived Infectability was significant (*B* = 0.101, *p* < 0.001). The 95% CI [0.030, 0.188] did not include zero and Z > 1.96, supporting the mediating role of Perceived Infectability. This path accounted for 23.71% of the total effect.

#### 3.4.2 Indirect effect via germ aversion (H3)

The indirect path from Nutrition-Related Food Neophobia to Food Disgust via Germ Aversion was significant (*B* = 0.132, *p* < 0.001). The 95% CI [0.067, 0.215] did not include zero and *Z* > 1.96, supporting the mediating role of Germ Aversion. This path accounted for 30.98% of the total effect.

#### 3.4.3 Total indirect effects

The sum of the effects of the two mediation pathways was 0.233. Its Bootstrap 95% confidence interval [0.141, 0.335] does not include 0 and *Z* > 1.96, indicating a significant total mediation effect. It explains 54.69% of the total effect. This indicated that more than half of the effect of “NRFN” on “FD” was mediated by the two internal psychological mechanisms: “PI” and “GA.”

## 4 Discussion

The present study investigated the structural relationships between Nutrition-Related Food Neophobia, Food Disgust (FD), and the mediating roles of the Behavioral Immune System (BIS)—specifically Perceived Infectability (PI) and Germ Aversion (GA)—among pre-service preschool teachers. Using an SEM approach, this study identified the psychological barriers that future “food gatekeepers” face when encountering novel nutritional interventions. Our findings support the premise that the rejection of biofortified or technologically modified foods is not merely a cognitive deficit in nutritional knowledge but is deeply rooted in evolutionary and socio-cultural mechanisms.

The structural model demonstrates that PI and GA collectively explain 54.69% of the total effect of NRFN on FD. Crucially, the mediating influence of Germ Aversion (30.98%) is numerically larger than that of Perceived Infectability (23.71%), revealing a distinctive psychological profile among health-trained future educators. These results provide a critical empirical foundation for the development of “Nutritional Health Literacy in Children and Adolescents” by identifying the primary behavioral determinants of those who curate the early childhood dietary environment.

### 4.1 The omnivore’s dilemma under compressed modernity: linking cognitive conflict to affective defense

Our results indicate that NRFN is significantly and positively associated with FD (H1), supporting the theoretical premise that nutritional novelty may elicit a visceral rejection response. This finding aligns with the “Omnivore’s Dilemma”—the evolutionary necessity to balance the drive for nutrient diversity against the risk of ingesting toxins. While traditional food neophobia research focused on sensory properties, our data supports the assertion by Razzaq et al. (8) that cognitive uncertainty surrounding a food’s nutritional profile or biofortification process acts as a potent trigger for neophobic reactions.

In the macro-context of “compressed modernity” (2), rapid technological changes in food systems can heighten cognitive uncertainty regarding nutritional profiles, which manifests as Nutrition-Related Food Neophobia. When future gatekeepers encounter hypothetical nutritional enhancements like biofortified rice—a staple deeply embedded in their cultural identity—this technological modification may disrupt their established “social space of food” (1) and safe-food schemas. Drawing on Rozin and Fallon’s (4) perspective on disgust, this study demonstrates that this cognitive unfamiliarity (NRFN) regarding nutritional traits is heuristically processed as a conceptual threat, which is strongly associated with an affective and physiological rejection response (FD).

Specifically, our findings tentatively suggest that under the pressure of ‘compressed modernity’, the technological ‘unnaturalness’ of biofortification is not merely processed as a cognitive uncertainty. Instead, it is filtered through the lens of highly institutionalized hygiene norms, which causes the novel food to be implicitly categorized as a biological threat. This process explains why Germ Aversion (GA) emerged as a more potent mediator than Perceived Infectability (PI): the cultural and technological friction in rapidly transitioning societies like China transforms a staple food’s nutritional upgrade into a perceived ‘contamination’ of its traditional and symbolic purity.

To further assess the empirical validity of the hypothesized directional pathways, an alternative structural model was tested based on the theoretical possibility that broader dispositional traits might precede specific food neophobia. In this alternative model, Perceived Infectability (PI) and Germ Aversion (GA) were specified as exogenous predictors, with Nutrition-Related Food Neophobia acting as the mediator, and Food Disgust (FD) as the outcome variable. While this alternative model demonstrated an acceptable absolute fit to the data (χ*^2^* = 735.191, df = 270, CFI = 0.937, RMSEA = 0.056), the non-nested model comparison using information criteria revealed a substantial statistical superiority for our original hypothesized model (χ*^2^* = 598.396, df = 270, CFI = 0.956, RMSEA = 0.047). The original model yielded notably lower values for both the Akaike Information Criterion (AIC = 708.396 vs. 845.191) and the Bayesian Information Criterion (BIC = 945.241 vs. 1082.036). In model selection, a ΔAIC greater than 10 indicates overwhelming empirical support for the superior model (26). Therefore, the pronounced differences (ΔAIC = 136.795, ΔBIC = 136.795) provide robust justification for our proposed configuration. This indicates that, within the context of compressed modernity, specific neophobic reactions to technologically altered foods are more optimally modelled as environmental triggers that activate latent behavioral immune responses, rather than merely acting as downstream products of general pathogen worry.

### 4.2 The behavioral immune system as a determinant: the GA-PI paradox in health-trained gatekeepers

A main theoretical contribution is the clarification of the Behavioral Immune System (BIS) as the underlying psychological mechanism of food rejection. The BIS is a set of psychological mechanisms that facilitate the avoidance of infection by preventing contact with and consumption of infectious microorganisms (10). Our findings indicated that both Perceived Infectability (PI) and Germ Aversion (GA) significantly mediate the path from NRFN to FD. However, the numerically larger indirect effect of GA (30.98%) compared to PI (23.71%) reveals a distinctive “paradox of education and intuition.”

This dual-pathway mediation lends empirical support to the foundational framework proposed by Airington et al. (6), who demonstrated that underlying disease avoidance motivations are closely intertwined with the behavioral expression of food neophobia. However, while Airington et al. (6) highlighted a generalized linkage between pathogen worry and food rejection, our findings extend this evolutionary paradigm into a highly specialized institutional domain. By contextualizing these mechanisms within pre-service preschool educators, our data reveal that this evolutionary disease-avoidance alliance is not uniform; rather, the affective component (GA) appears numerically larger than the cognitive assessment (PI). This shift suggests that cultural and professional socialization under compressed modernity can amplify specific evolutionary aversion nodes, transforming a general adaptive trait into an acute psychological barrier against nutritional innovation.

Conceptually, the BIS consists of two distinct dimensions that predict different types of psychological and behavioral responses (11). Perceived Infectability (PI) represents a cognitive-rational assessment of one’s own immunological vulnerability, predicting responses informed by more rational, cognitive appraisals of susceptibility (11, 27). In contrast, Germ Aversion (GA) is an affective-evolutionary response, predicting reactions rooted in intuitive emotional appraisals toward external pathogen cues (11). The pre-service teachers in our sample have undergone rigorous, high-stakes training in ‘Preschool Child Hygiene’ and ‘Infectious Disease Prevention.’ While the current study did not compare these students with a non-health-trained control group, previous literature suggests that rigorous hygiene norms can inadvertently ‘prime’ the affective component of the BIS (28).

This phenomenon can be explained by the professional exigencies of the teaching environment. When hygiene norms are framed as rigid moral and professional mandates, any food item that deviates from a traditional, “sterile,” or “natural” state (i.e., biofortified rice) is heuristically processed as a potential contamination threat (12). Research in evolutionary psychology suggests that deviations from familiar food states act as reminders of pathogen risks, generating immediate disgust to motivate strict compliance with disease-avoidance (4, 12). Disgust, in this context, serves as a powerful signal to avoid potential sources of infection, a mechanism that strongly supported the survival of our ancestors in pathogen-rich environments (29).

This “hyper-vigilance” transforms professional knowledge into a visceral emotional barrier. As a result, the intuitive, affective disgust triggered by germ aversion (GA) becomes a more immediate and potent predictor of food rejection than the more abstract, cognitive self-assessment of disease susceptibility (PI) (11). This suggests that the rigorous hygiene training intended to protect children may paradoxically create a sanitization-driven phobia that severely restricts the gatekeeper’s psychological acceptance of nutritional innovations (1, 28).

### 4.3 The kindergarten as a social space: implications for implementation fidelity

These psychological barriers in future preschool teachers have significant implications for the “Educational Tools” and “Behavioral Determinants” central to children’s nutritional health. Lewin’s (30) gatekeeper theory emphasizes that individuals control the channels through which food reaches the table. In the modern educational landscape, preschool teachers have extended this role, acting as the primary curators of the children’s micro-dietary environment.

In the kindergarten, the dining table functions as a “social space of food” where children internalize dietary norms through social modeling. Children are highly sensitive to the affective responses of their adult gatekeepers. If a teacher harbors implicit Food Disgust driven by their own Germ Aversion toward a novel biofortified food, they may exhibit subtle, non-verbal cues of avoidance or reluctance. According to social modeling theory, children internalize these cues as signals of danger, thereby adopting the gatekeeper’s neophobia. This psychological resistance in teachers could act as a “structural determinant,” which may contribute to “implementation fidelity loss” for public health interventions. Even the most scientifically advanced educational tools—such as sustainable diet modules or biofortification programs—might be less effective if the gatekeepers executing them harbor deep-seated, unaddressed psychological barriers.

### 4.4 Paradigm shift: upstream preventive and educational strategies

Our data supports a reevaluation of current strategies for improving nutritional health literacy in younger populations: preventive nutrition must be an “upstream” intervention starting with the psychological resilience of the gatekeepers. Identifying that formal cognitive training alone cannot dismantle evolutionary disease-avoidance mechanisms reveals a critical gap in current teacher-training programs.

#### 4.4.1 Proposed training framework for pre-service teachers

Translating these insights into practice suggests the potential value of a multi-dimensional training framework designed for teacher-training curricula. Based on our findings, we tentatively propose a framework focusing on cognitive reframing to potentially mitigate germ aversion:

Module 1: The Evolutionary Psychology of Food Choice. This module could educate future teachers on the origins of disgust. By understanding that their aversion to “unnatural” food forms is an over-active biological alarm rather than a factual safety risk, teachers can achieve a “meta-cognitive” distance from their instinctive rejection.

Module 2: Cognitive Reframing of “Naturalness” and “Safety.” Utilizing “Evidence-Based Reassurance” techniques, this module might help reframe the “technological intervention” not as a “contamination” but as a “hygiene optimization,” thereby aligning the innovation with the teacher’s professional hygiene values.

Module 3: Gatekeeper Social Modeling Protocols. This practical module could use role-playing to train teachers in “Enthusiastic Modeling,” ensuring they do not inadvertently transmit subtle “disgust signals” to children within the social space of food.

### 4.5 Beyond downstream interventions: the significance of the gatekeeper paradigm shift

For decades, the literature on pediatric nutrition has emphasized ‘child-centered’ interventions. For instance, the landmark studies by Birch (31) and Cooke (32) have proven that repeated exposure and positive reinforcement can reduce children’s neophobia. However, these ‘downstream’ approaches often operate under the assumption that the food environment provided by adults is neutral. Our findings challenge this assumption by demonstrating that future gatekeepers harbor significant, evolution-based psychological barriers (GA and PI) that act as a ‘distorting lens’ for any nutritional intervention.

This suggests a necessary paradigm shift in preventive nutrition. Rather than focusing solely on ‘fixing the child,’ effective interventions must prioritize ‘desensitizing the gatekeeper.’ If a teacher utilizes an educational tool while internally experiencing germ-driven disgust, the ‘social modeling’ effect—a cornerstone of child development research—becomes a vehicle for transmitting neophobia rather than curiosity. Therefore, shifting the intervention focus ‘upstream’ to the pre-service training of teachers represents a more structurally sound approach. By addressing the gatekeepers’ germ aversion, we secure the foundational integrity of the kindergarten’s social space of food, ensuring that downstream child-centered tools can function in a truly supportive and neophobia-free environment.

### 4.6 Limitations and future research

This study utilized a sample of 548 participants, exceeding the statistical power requirements for complex SEM models (16). However, its cross-sectional design limits absolute causal inferences. Second, while Harman’s single-factor test provided a preliminary diagnostic suggesting that common method bias (CMB) was not pervasive, it does not definitively rule out CMB. The inherent risk of method variance in self-reported cross-sectional data remains a potential limitation. Furthermore, the reliance on a purposive sample of pre-service teachers from a single university in Changsha limits the broad generalizability of our findings. Therefore, our interpretations regarding actual classroom food environments and children’s dietary behaviors should be viewed as theoretical implications that require future empirical validation among in-service professionals across diverse geographical regions. Additionally, the phrasing ‘techniques’ used to describe the biofortified rice stimulus may have inadvertently conflated general nutritional enhancement with genetic modification concerns, an aspect that future stimulus designs should carefully disentangle. Future research should employ longitudinal designs to observe how these psychological barriers fluctuate as pre-service teachers transition into professional practice. Third, a notable limitation concerns the potential constraint on the construct validity of our stimulus material. In this study, we utilized ‘biofortified rice’ as the specific visual stimulus to elicit NRFN responses, while utilizing the FDS to capture a general, trait-like propensity for food disgust. While rice was strategically chosen as a culturally foundational staple to probe the ‘social space of food’ within the Southern Chinese context, the psychological reactions observed may be specific to staple crop interventions. Pre-service teachers’ perceptions of ‘pathogen risk’ or ‘unnaturalness’ might fluctuate significantly when presented with different categories of nutritional innovation, such as biofortified vegetables, functional snacks, or more technologically radical meat-alternatives. Consequently, the reliance on a single food category may not fully capture the spectrum of neophobic responses across the broader landscape of nutrition-related novel foods. Future research should employ a diverse array of multi-category stimulus materials to validate whether these behavioral determinants remain consistent across varied food groups and degrees of technological processing. Finally, experimental research could test whether specific sensory-focused interventions or “cognitive reframing” tools can effectively down-regulate Germ Aversion among educators, providing a clearer operational guide for future preventive nutrition policies.

## 5 Conclusion

This study demonstrates that Nutrition-Related Food Neophobia is significantly associated with Food Disgust (FD) among pre-service preschool teachers, a relationship primarily mediated by evolutionary disease-avoidance mechanisms. Notably, Germ Aversion (GA) shows a numerically larger mediating influence than Perceived Infectability (PI), highlighting that the rigorous hygiene training of future educators may inadvertently heighten their affective resistance to novel nutritional foods.

These results provide critical insights for the “Behavioral Determinants” of pediatric nutrition. The identified psychological barriers may threaten the implementation fidelity of “Educational Tools” and the cultivation of a diverse “social space of food” in kindergartens. To successfully improve children’s nutritional health literacy, “Preventive Strategies” should consider shifting upstream to address the psychological resilience of future food gatekeepers. Future policies could consider implementing specific training modules—such as evolutionary psychology deconstruction and cognitive reframing of food technology—to dismantle the germ aversion of adult food gatekeepers and potentially improve the implementation fidelity of pediatric nutritional interventions. These findings highlight the need to shift from traditional downstream, child-centered interventions to an upstream, gatekeeper-focused approach. This transition recognizes that the psychological resilience of preschool teachers might act as a key behavioral determinant of children’s nutritional health literacy. Future preventive strategies should therefore prioritize the psychological deconstruction of evolutionary barriers in educators to ensure the successful cultivation of healthy eating habits in the next generation. Given that our findings are based on a purposive sample of pre-service teachers, future longitudinal studies among in-service professionals are needed to validate how these psychological barriers translate into actual classroom food environments and children’s dietary behaviors.

## Data Availability

The original contributions presented in the study are included in the article/Supplementary material, further inquiries can be directed to the corresponding author/s.
