# Supplementary material for: Psychological barriers in future food gatekeepers: How germ aversion and perceived infectability mediate nutrition-related food neophobia among pre-service preschool teachers
Source: Front Nutr. 2026 Jul 10;13:1863520. doi: 10.3389/fnut.2026.1863520 (PMC13397584; doi:10.3389/fnut.2026.1863520)
Supplement: Supplementary file 2 [file Table_1.DOCX]

**Appendix S1 Table.** Scale English- Chinese Correspondence

| **English** | **Chinese** |
| --- | --- |
| *Nutrition-related Food Neophobia (NRFN)* (1) |  |
| I am afraid that this rice will harm my health. | 我担心这种米饭会危害我的健康。 |
| I will try this rice only after I have made sure it is safe for consumption. | 我会在确保这种米饭安全可食用之后再尝试它。 |
| I am not willing to try this rice because I think ordinary rice has all the micronutrients I need. | 我不愿意尝试这种米饭，因为我认为普通米饭已经包含了所有我需要的微量元素。 |
| I am willing to try this rice even if that makes me feel uncomfortable. [R] | 我愿意尝试这种米饭，即使这让我感到不舒服。[R] |
| I am not willing to try this rice because I fear that more than the naturally occurring amount of micronutrient in rice is not suitable for my body. | 我不愿意尝试这种大米，因为我担心其中的微量营养素含量超过我身体所能承受的自然水平。 |
| *Perceived Infectability (PI)* (2) |  |
| If an illness is ‘going around’, I will get it. | 如果一种疾病正在“流传”，我就会得这种病。 |
| My past experiences make me believe I am not likely to get sick even when my friends are sick. [R] | 我的过去经历让我相信，即使我的朋友生病了，我也不太可能生病。[R] |
| I have a history of susceptibility to infectious disease. | 我有感染传染病的易感史。 |
| In general, I am very susceptible to colds, flu and other infectious diseases. | 总的来说，我很容易感冒、流感和其他传染病。 |
| I am more likely than the people around me to catch an infectious disease. | 我比周围的人更容易感染传染病。 |
| I am unlikely to catch a cold, flu or other illness, even if it is ‘going around’. [R] | 即使感冒、流感或其他疾病在传播，我也不太可能感染。[R] |
| My immune system protects me from most illnesses that other people get. [R] | 我的免疫系统能保护我免受大多数人会得的疾病。[R] |
| *Germ Aversion (GA)* (2) |  |
| It really bothers me when people sneeze without covering their mouths. | 当人们打喷嚏时不捂住嘴巴真的让我很烦。 |
| I am comfortable sharing a water bottle with a friend. [R] | 我愿意和朋友共用同一个水瓶喝水。[R] |
| I do not like to write with a pencil someone else has obviously chewed on. | 我不喜欢用别人咬过的铅笔写字。 |
| I prefer to wash my hands pretty soon after shaking someone’s hand. | 我更习惯在和别人握手后马上洗手。 |
| I dislike wearing used clothes because you do not know what the last person who wore it was like. | 我不喜欢穿别人穿过的衣服，因为不知道上一个穿它的人是什么样的人。 |
| My hands do not feel dirty after touching money. [R] | 我的手摸钱后不觉得脏。[R] |
| It does not make me anxious to be around sick people. [R] | 和生病的人在一起不会让我感到焦虑。[R] |
| *Food Disgust (FD)* (3) |  |
| To see raw meat. | 当我看到生肉。 |
| To eat with dirty silverware in a restaurant. | 在餐馆里用脏的餐具吃饭。 |
| A meal prepared by a cook who has greasy hair and dirty fingernails. | 我对园长的管理方式感到满意。 |
| If the cook in a restaurant has an open cut. | 一顿由一个头发油腻、指甲脏乱的厨师准备的饭菜。 |
| To eat raw fish like sushi. | 生吃鱼肉，比如寿司。 |
| Food donated from a neighbor whom I barely know. | 一个不认识的邻居赠送给我的食物。 |

Note: NRFN = Nutrition-related Food Neophobia; PI = Perceived Infectability; GA = Germ Aversion; FD = Food Disgust. [R] = indicates reverse score items.

**REFERENCES**

1. Razzaq A, Tang Y, Qing P. Towards Sustainable Diets: Understanding the Cognitive Mechanism of Consumer Acceptance of Biofortified Foods and the Role of Nutrition Information. *IJERPH* (2021) 18:1175. doi: 10.3390/ijerph18031175

2. Liu W, Li L, Han Y, Li Y. Effect of perceived vulnerability to disease on anxiety: The mediating role of certainty in control and moderating role of social support (In Chinese). *China Journal of Health Psychology* (2021) 29:1688–1693. doi: 10.13342/j.cnki.cjhp.2021.11.019

3. Zhang L, Wu Y, Jin T, Jia Y. Reliability and validity of Chinese short version of the food disgust scale applied in Chinese college students (In Chinese). *Modern Preventive Medicine* (2021) 48:577–579, 598.
